# Institutional objection to abortion: A mixed-methods narrative review

**DOI:** 10.1177/17455057231152373

**Published:** 2023-02-13

**Authors:** Bronwen Merner, Casey M Haining, Lindy Willmott, Julian Savulescu, Louise A Keogh

**Affiliations:** 1 Centre for Health Equity, Melbourne School of Population and Global Health, The University of Melbourne, Parkville, VIC, Australia; 2 Australian Centre for Health Law Research, Faculty of Business and Law, Queensland University of Technology, Brisbane, QLD, Australia; 3 The Oxford Uehiro Centre for Practical Ethics, University of Oxford, Oxford, UK; 4 Centre for Biomedical Ethics, Yong Loo Lin School of Medicine, National University of Singapore, Singapore, Singapore; 5 Murdoch Children’s Research Institute, Parkville, VIC, Australia; 6 The University of Melbourne, Parkville, VIC, Australia

**Keywords:** abortion, conscientious objection, institutional conscientious objection, institutional objection, religious hospitals

## Abstract

Institutional objection (IO) occurs when institutions providing health care claim objector status and refuse to provide legally permissible health services such as abortion. IO may be regulated by sources including law, ethical codes and policies (including State and local/institutional policies). We conducted a mixed-methods narrative review of the empirical evidence exploring IO to abortion provision globally, to inform areas for further research. MEDLINE (Ovid), Embase (Ovid), CINAHL (EBSCO), Global Health (CAB Abstracts), ScienceDirect and Scopus were searched in August 2021 using keywords including ‘conscientious objection’, ‘faith-based organizations’, ‘religious hospitals’ and ‘abortion’. Eligible research focused on clinicians’ attitudes and experiences of IO to abortion. The 28 studies included in the review were from nine countries: United States (19), Chile (2), Turkey (1), Argentina (1), Australia (1), Colombia (1), Ghana (1), Poland (1) and South Africa (1). The analysis demonstrated that IO was claimed in a range of countries, despite different legislative and policy frameworks. There was strong evidence from the United States that clinicians in religious healthcare institutions were less likely to provide abortions and abortion referrals, and that training of future abortion providers was negatively affected by IO. Qualitative evidence from other countries showed that IO was claimed by secular as well as religious institutions, and individual conscientious objection could be used as a mechanism for imposing IO. Further research is needed to explore whether IO is morally justified, how decisions are made to claim IO, and on what grounds. Finally, appropriate models for regulating IO are needed to ensure the protection of women’s access to abortion. Such models could be informed by those used to regulate IO in other contexts, such as voluntary assisted dying.

## Introduction

In health care, individual conscientious objection (CO) arises when health practitioners refuse to be involved in providing guidance or treatment to certain patients due to their moral, religious or philosophical beliefs.^1^ Allowing health practitioners to conscientiously object to legally available health services, such as abortion, aims to protect their moral integrity.^2^ Wicclair argues that healthcare institutions should aim to protect the moral integrity of health practitioners without significantly compromising other important values and interests.^3^ However, there is evidence that CO is misused by some health practitioners to refuse participation in abortion provision for reasons other than conscience.^4–6^ There are several consequences of individual CO on abortion access including a reduction in the pool of willing abortion providers, delays in women accessing abortion and exacerbation of the stigma associated with abortion.^1^

Institutional objection (IO) occurs when institutions providing health care claim objector status and refuse to provide legally-permissible care, such as abortion.^4^ Similar to individual CO, IO seeks to preserve institutional integrity by allowing hospitals to refuse to provide care that would undermine their mission and identity.^2^ Healthcare institutions may also refuse to provide legally-permissible care for reasons other than IO. For example, a hospital may refuse to provide abortions because it does not have adequate numbers of trained staff, or lacks the facilities required to provide the care safely.^7^ However, in this article, we focus on hospitals holding an IO to providing abortion services.

The claim that hospitals, rather than individuals, have and can exercise a conscience is contested.^2,8,9^ Some philosophers argue that hospitals are physical buildings and therefore cannot be autonomous moral agents.^9^ They contend that recognizing institutional conscience erodes the protection of the individual consciences of those employees whose moral stance differs from the institution.^9^ In contrast, other philosophers have argued that corporations (and by extension health institutions) can be morally responsible agents which have moral identities and consciences.^10,11^

Even if institutions have an interest in protecting their moral integrity and identity, this is subject to limits.^2^ Institutions delivering health care have general obligations to protect their patients from harm, to promote their health and respect their autonomy.^2^ Thus, when pregnant people experience complications that may chance or require termination of a pregnancy (such as pre-eclampsia, ectopic pregnancy and incomplete miscarriage), objecting institutions have ethical obligations to explain all relevant clinical options (including those they do not offer) and, time-permitting, offer a transfer to a nonobjecting institution.^2^ Further to this, when time does not permit a transfer, and withholding emergency treatment would expose the patient to an excessive increased risk of harm, the institution has an obligation to offer the treatment.^2^ However, evidence suggests the management of these obligations varies in practice.^12,13^

Regulation of IO can take many forms. Regulation may include law which often has strong coercive force, but also comparatively weaker forms of regulation such as policies (including State and institutional policies) and ethical codes/directives.^14^ There is also evidence of ‘unwritten’ policies regulating IO to abortion, for example, hospital directors refusing to allow their staff to perform abortions.^15^ Ethical codes/directives also exist that have a regulatory influence across multiple health institutions. For example, Catholic policies and directives often seek to ensure that health care is delivered in a manner which accords with the Catholic ethos including respect for unborn human life.^16,17^ This is reflected in the *Ethical and Religious Directives for Catholic Health Care Services* (ERD) which prohibits the provision of abortion on request in US hospitals. It also bans health employees from taking ‘direct action’ against the embryo in ectopic pregnancy management.^16^ For pregnancy complications, a treatment that may endanger the foetus is only permitted when risks to the woman’s health or life are comparable to the risks posed to the foetus, and when harm to the foetus is not the intended goal of the treatment.^16^

Research from the United States has shown that women generally do not necessarily anticipate or support differences between Catholic and non-Catholic hospitals with respect to the provision of reproductive services (including abortion and miscarriage management). A nationally-representative survey of 2857 women demonstrated that most women did not support allowing hospitals to restrict reproductive care on the basis of religion.^18^ Although the study showed women were less likely to expect an abortion for foetal indications from a Catholic than a non-Catholic hospital, women in the lowest income group were less likely to identify a hospital as Catholic.^19^ Another study, consisting of a convenience sample of 236 reproductive-aged women, showed that participants expected obstetricians and gynaecologists (OBGYN) to provide family planning services, including abortion, regardless of institutional affiliation.^20^ Together, these findings suggest that in the United States, IO is not widely understood or supported by women.

Evidence about the impact of IO on patient outcomes is limited. A recent scoping review examined reproductive health care provision and patient outcomes in Catholic health facilities in the United States.^21^ The review included any type of family planning service, management of miscarriage or ectopic pregnancy, and infertility management. The authors concluded that little is known about reproductive health outcomes in Catholic facilities, which is particularly concerning given their prevalence in the US health system.

Fiala and Arthur^22^ have argued that the consequences of IO may be worse for abortion access than individual CO. They suggest IO may reduce the pool of abortion providers because otherwise willing health practitioners are unable to provide abortions within some religious hospitals (particularly Catholic hospitals) due to their institution’s position. They also assert IO may result in women seeking abortions having to travel further and pay more for the procedure, further disadvantaging those from marginalized populations. Women may also require more complex and risky abortion procedures at a later gestational stage if their access is delayed.^22,23^ Furthermore, the training of future health providers may be limited if IO results in less abortion training.^22^ While empirical research on individual CO is growing,^24,25^ IO has been less studied.^22^

To date, a formal literature review of existing global empirical evidence on IO to abortion has not been conducted. This mixed-methods narrative review aims to fill that gap by critically examining the empirical research about IO to abortion. We sought to understand the following:

- In which countries is IO being claimed?
- Which types of hospitals claim IO?
- How are decisions made about claiming an IO?
- How is IO regulated in different countries?

## Body

### Methods

We conducted a mixed-methods narrative review of qualitative, quantitative and mixed methods studies exploring IO to abortion. We chose a narrative rather than a systematic review because a narrative review is less methods-driven allowing more scope for interpretation and critique of the evidence.^26^ Given the authorship team comprised international experts across women’s health, health sociology, ethics and law, such scope was warranted. In addition, the evidence for IO was sparse (except in the United States), therefore more flexibility in the literature searching was required.^27^

Initial database searches of MEDLINE (Ovid), Embase (Ovid), CINAHL (EBSCO), Global Health (CAB Abstracts), ScienceDirect and Scopus were conducted in August 2021 using keywords including ‘conscientious objection’, ‘abortion’ and ‘termination’. These searches identified many studies on conscientious objection; however, reference list searches of included references showed that the search was not identifying more detailed literature about IO. Thus, a further database search was conducted, also in August 2021, using keywords such as ‘faith-based organizations’, ‘religious hospitals’ and ‘abortion’. For pragmatic reasons, the search was limited to English language articles only, and publication from January 2000 to August 2021.

Studies were selected for eligibility based on the inclusion and exclusion criteria shown in Table 1. For the purposes of this review, we included studies in which IO to abortion was occurring in practice, regardless of how IO was regulated. This criterion allowed us to capture a wider range of IO.

**Table 1.** Inclusion and exclusion criteria.

	Inclusion criteria	Exclusion criteria
Populations	Healthcare practitioners and student healthcare practitioners	Non-healthcare practitioners (e.g. hospital chaplains)
Context	Abortion only or abortion included as part of family planning/reproductive healthcare	Reproductive health care or family planning that did not include abortion (e.g. studies specifically on contraception or reproductive assistive technologies)
Phenomenon	Institutional objection	Focuses solely on individual conscientious objection
Study types	Empirical primary qualitative, quantitative and mixed methods peer-reviewed journal articles	Letters, editorials, conference abstracts and posters, nonempirical research, systematic or literature reviews

The reference lists of included studies as well as relevant systematic or literature reviews were also checked for eligible references.

Following the selection of the included studies, study details (e.g. author/s, title, date of publication, and country) and legal context (including legal status of abortion and IO) were extracted and summarized. The studies were then categorized according to country/region given the legal context of abortion and regulation of IO varied. The findings of individual studies and authors’ interpretations relevant to IO were then extracted and analysed using thematic analysis. Where appropriate, methods were critiqued.

### Results

After the removal of duplicates, database searches yielded a total of 1100 references. After title and abstract screening, 68 articles remained for full-text screening. Of those, 28 met the inclusion criteria. Included studies were from nine countries: United States (19), Chile (2), Turkey (1), Argentina (1), Australia (1), Colombia (1), Ghana (1), Poland (1) and South Africa (1). Each of the studies is analysed below, grouped geographically.

#### North America

Many of the empirical studies about IO to abortion in the United States focused on miscarriage management in Catholic hospitals.^13,28–31^ The studies were conducted in the context of Catholic hospitals increasing their market share of health services and concerns about how this may restrict access to reproductive health care.^32^ Using a mystery caller approach, researchers (acting as patients) called 144 Catholic-owned or Catholic-affiliated clinics in the United States and requested appointments for birth control, tubal ligation and abortion. The results showed only 2% of the clinics would offer an abortion for an unwanted pregnancy.^29^ A limitation of this study was that the person answering the phone may not have been aware of the clinic’s relevant policies.

In the United States, large cross-sectional surveys have been used to quantify the impact of IO on individual clinical practice. A cross-sectional survey examining abortion provision was sent to a nationally representative sample of 1800 OBGYN with a high response rate of 66%. The results showed that working primarily in a Catholic institution was associated with a decreased likelihood of abortion provision (odds ratio [OR] = 0.32, 95% CI = 0.16–0.68).^33^ The same survey also demonstrated that 37% of OBGYN working in religiously affiliated facilities had experienced conflict with the institution about religiously-based policies. Conflicts were more common in Catholic facilities than other faith-based institutions.^34^ Another survey of a nationally-representative sample of 3000 primary care physicians about pregnancy options, counselling and abortion referrals demonstrated that working at a Catholic institution was significantly associated with lower odds of routine referral (OR 0.27 95% CI 0.11–0.66).^35^ The survey response rate was 29%. An earlier survey of 879 primary care physicians about their experiences with conflict about patient care in religious institutions yielded a 52% response rate. The results showed that of the 43% who had worked in a religious institution, 19% had experienced conflict related to religiously-based policies.^36^ A national sample of 2125 maternal-foetal medicine subspecialists were surveyed about barriers to dilation and evacuation (D&E) practice. The survey included both quantitative and qualitative components and the response rate was 32%. Qualitative results showed some participants described religious institutional restrictions (such as inability to perform elective terminations) as a barrier to D&E. Quantitative results showed the biggest barrier reported by D&E providers (37%) was a negative culture comprising unsupportive staff or colleagues, institutional restrictions and concerns for personal safety.^37^

Qualitative studies have provided in-depth data on the experiences of clinicians working in institutions objecting to abortion. In multiple studies, OBGYN and other physicians in Catholic hospitals perceived they were unable to provide standard clinical management for pregnancy complications due to mandated restrictions.^12,28,30,38–40^ Examples of restrictions included being unable to use methotrexate (a drug used for medical abortions), needing to ascertain and document the nonviability of the foetus before performing a medically indicated abortion, and being unable to provide tubal ligation at the same time as managing ectopic pregnancy. Three studies also found that clinicians’ miscarriage management decisions were delayed or impeded because they needed approval from the hospital’s ethics committee.^12,31,40^ Catholic hospitals’ ethics committees are expected to abide by the ERD when helping clinicians resolve ethical challenges.^41^

Some health practitioners who were unable to perform an abortion due to their workplace’s IO referred or transferred the patient to a nonobjecting hospital where the procedure could be completed. Several qualitative studies have explored issues associated with referral and/or transfer of patients seeking abortions to nonreligious hospitals.^12,13,28,38,40,42^ Some providers in these studies felt conflicted about transferring patients because it could cause treatment delays,^40^ interrupt continuity of care^12^ or, in the case of referring patients with a wanted pregnancy to an abortion clinic, be perceived as an unnecessary punishment.^42^ Direct referrals for abortions could be impeded by nurses or office staff refusing to facilitate the referral^13^ or the prerequisite of getting the ethics committee’s approval.^12^

Surveys have been used to investigate the implications of IO on future abortion providers in the United States. A recent survey sampled all OBGYN residency training programmes in the United States (79% response rate) and found no difference in the availability of abortion training between nonreligious and religiously affiliated institutions.^43^ These counterintuitive results were explained by the authors who indicated that religiously affiliated hospitals were partnering with secular hospitals to meet abortion training requirements, but noted that further research was needed. Programme directors were also asked about restrictions on abortion training within their institutions. Examples of restrictions included those imposed by restrictive hospital policies, law or resistance from nursing staff. Directors in religiously affiliated institutions reported there were more restrictions on abortion training than nonreligiously affiliated institutions.^44^ A survey of 454 programme directors and chief residents at US family medicine residencies (54% response rate) found religiously affiliated institutions were significantly less likely to offer routine abortion training (*p* = .041).^45^ In another study, 30 programme leaders for OBGYN residency programmes at Catholic and other religious hospitals (e.g. Lutheran, Methodist facilities) were surveyed about family planning training. The results showed Catholic hospitals were more likely to report poor abortion training compared to other religious hospitals (47% versus 0%, *p* = .04).^46^ Surveys also showed many medical students^47^ and medical residency programme directors^46^ in religiously affiliated institutions expressed concern or dissatisfaction with training limitations. A qualitative study of 31 OBGYN, who trained in religiously affiliated hospitals, reported that many participants perceived that religious policies had negatively impacted their training experiences and put limitations on the range of reproductive health services they could now provide.^48^

#### Latin America

In 2017, Chile moved from a complete ban on abortion to legally permitting the procedure in restricted circumstances.^49^ As well as protecting individual CO, Chile’s abortion law also protected the right of private institutions (including those receiving public funding) to claim objector status.^49^ Public institutions were prohibited from claiming IO.^49^ The highest-ranking university and medical school in Chile, the Pontificia Universidad Católica de Chile (a private Catholic university), was the first institution to claim objector status prompting concerns among its medical students.^49^ In an interview study of 30 medical and midwifery faculty members, including 10 from religious universities and 20 from secular universities, the data indicated that all of the religious faculty (and none of the secular faculty) supported the right to claim IO.^49^ Though this suggests support from religious faculty for IO, the sample size was small and therefore may not reflect the views of the wider religious faculty. A cross-sectional survey of 333 medical and midwifery students across four secular and three religious universities in Santiago demonstrated around 50% of students at religious universities and 20% at secular universities supported IO.^50^ The authors stated these findings suggested a mismatch between the administration of religious institutions, which staunchly supported and claimed IO, and their students. They reported that Chile’s Catholic universities were some of the most prestigious in the country and thus attracted students irrespective of their religious views.^50^ While this study included students from a range of universities, 77% of respondents attended secular universities. The authors stated the underrepresentation of respondents from religious universities likely underrepresented views supportive of conscience-based objections.^50^

The abortion laws in Argentina were liberalized in 2020.^51^ Prior to liberalization, abortion was legally restricted and regulations issued by the Argentine National Congress acknowledged that both private and public institutions possessed a right to IO.^52^ In a cross-sectional survey of sexual and reproductive health providers in Argentina’s public health system, 38% of respondents (n = 269) believed some of their colleagues claimed objector status following mandates from their managers or heads of department.^52^ In the qualitative component, 11 nonobjector heads of reproductive health programmes and health departments in Argentina were interviewed about CO to abortion.^52^ Some of the participants perceived that ‘hospital authorities (such as directors, chiefs of service and faculty) have used CO to establish an ideological approach to sexual and reproductive health care in their departments’.^52 p.274^ For instance, one of the participants stated, ‘In hospitals you find people saying “the CO [form] must be signed” and those orders came from department heads’.^52 p.274^ A limitation of this study was the recruitment of participants was largely through pro-choice networks, likely oversampling abortion providers. However, the presence of IO as a problem in both the survey and qualitative results strengthens these findings.

Abortion in Colombia was decriminalized in 2022.^51^ Even prior to decriminalization, institutions were prohibited by case law from objecting to abortion provision.^53^ In a 2016 study of CO to abortion in Bogota, interviews with 13 key informants from all sides of the abortion debate reported that some religious hospitals were claiming IO despite the legal prohibition from doing so.^53^ Furthermore, the authors reported: ‘A physician who worked in one of the implicated institutions explained that she and her colleagues were asked to “voluntarily” sign declarations of objection when they began their jobs at the hospital’.^53 p.74^ Given the key informants were not all clinicians (and included not-for-profit leaders, lawyers, women’s rights advocates, bioethicists, a government official and a professor of medicine), their ability to provide direct experiences of IO may have been limited. However, the key informants may also have had a broader overview of systemic and policy issues than individual clinicians.

#### Africa

In South Africa, abortion was decriminalized under the *Choice on Termination of Pregnancy Act 1996* (CTOPA).^54^ Under CTOPA guidelines, Favier, Greenberg^54^ reported that CO was restricted to individual clinicians, and to the actual abortion procedure.^54^ However, their case study, which included interviews with nine key informants (including medical practitioners, government officials and nongovernment organization staff) and a desk-based literature review,^54^ showed that public health facilities in rural or conservative regions became de facto institutional objectors when sufficient practitioners refused to provide abortions.^54^ Similar to the studies in Argentina and Bogota, the authors reported certain health facilities asked newly-employed staff to sign individual CO forms:

Interviewees described gaps between regulation and service delivery, with one interviewee reporting that certain facilities give all new hires a conscientious objection ‘form letter’ to sign, an approach at odds with the ostensible purpose of permitting an individual choice with a deeply-felt rationale specific to each objector.^54^

Key informants also perceived there was a lack of government action against facilities claiming IO:

Several interviewees said that many facilities had become de facto institutional ‘objectors’, and that the [Department of Health] ‘should actually go to the facility and reprimand the facility manager’. However, one interviewee expressed doubt that the [Department of Health] had disciplinary purview over these recalcitrant facilities, and others described little enforcement of measures that did exist.^54 p.42^

This study had a small sample size but the similarities of their findings to studies in other countries strengthen confidence in their results.

In Ghana, abortion is legally restricted and only lawful when it is necessary to save a woman’s life, for physical or mental health reasons, in cases where the pregnancy resulted from rape or incest, and in cases of foetal impairment.^55^ Awoonor-Williams, Baffoe^55^ have reported that abortion services were provided within regional and district public health facilities in Ghana’s Eastern and Volta regions. Religiously affiliated private hospitals were governed by protocols that discouraged access to health care that prevented conception and childbirth.^55^ The qualitative interview and focus group study of 14 doctors and 20 midwives found that Catholic hospitals put additional restrictions on abortion provision beyond those set out in law. Two midwives working at Catholic hospitals reported that abortions were only performed to save a woman’s life or in the case of foetal impairment, but not to protect a woman’s physical or mental health nor in cases of rape or incest.^55^ One of the strengths of this study was that recruitment occurred via purposive sampling of regional and district hospitals, rather than via pro-choice networks. Participation was voluntary; however, there may still have been some self-selection bias towards those with stronger pro- or anti-abortion views. The firsthand accounts of IO in religious hospitals suggested the practice did occur; however, the findings cannot be generalized to other religious hospitals in Ghana.

#### Eastern Europe

In Poland, abortion is heavily restricted with the procedure only being allowed when the woman’s health is in danger or the pregnancy is a result of rape or incest.^56^ An ethnographic study^57^ explored how an individual CO clause, enshrined in the Medical Code of Ethics, served to further limit the narrow range of abortions available. The authors found that the clause contributed to a large scale denial of abortion when hospital leaders who were against abortion declared the procedure would not be conducted in their hospitals. In addition, employees holding opposing views to management risked losing their jobs if they spoke out.^57^ In 2003, a statement was issued by the Minister for Health clarifying that IO was not permitted; however, the authors reported that no court action had been instigated for violations of the individual CO clause. This rigorous study involved 19 months of fieldwork across Warsaw, Krakow and Gdansk, including participant-observation and interviews with 123 women, 26 physicians specializing in OBGYN and six family planning instructors in Warsaw and Gdansk.

#### Middle East

In Turkey, abortion is legally available on request in the first 10 weeks of pregnancy with no legal right to individual CO.^58^ Although the Turkish Ministry of Health claimed that all state hospitals with obstetrics and gynaecology services performed abortions, a mystery caller study in 2017 found only 7.8% provided abortion without regard to reason.^59^ The percentage of state teaching hospitals providing abortion on request was slightly higher at 15.5%. The author found that women in rural areas were less likely to have access to abortion at state hospitals without restriction than those in metropolitan areas. The author was also concerned about the future of abortion provision in Turkey, given only a small percentage of state teaching hospitals engaged in abortion provision as provided for by law.^59^

In the same study, O’Neil found the percentage of state hospitals in Istanbul providing abortion on request was higher than the overall country percentage (14% compared to 7.8%).^58^ However, the author concluded that IO at state hospitals in Istanbul was resulting in a de facto privatization of abortion services, where inflated prices resulted in fewer women being able to afford the procedure.^58^ A strength of this study was the mystery caller approach which may have provided a realistic insight into the patient experience. However, a potential limitation of this method was that some of the hospital staff contacted may not have been fully informed about abortion procedures at the hospital.

#### Oceania

In Australia, the state of Victoria decriminalized abortion in 2008. In Victoria, individual CO to abortion is explicitly protected by law, but IO is not. In a qualitative interview study about individual CO protection with 19 experts in abortion provision, some participants reported concerns religious hospitals were claiming IO.^60^ However, as IO was not the focus of the study, these perceptions were not explored in-depth.

### Discussion

This review of the empirical research about IO to abortion raises important philosophical and practical questions. First, the findings demonstrated that hospitals across the world were claiming IO to refuse to provide abortions. Such claims were based on a contested premise that, like individuals, institutions can be autonomous moral agents with moral responsibilities. The review identified strong survey evidence from the United States that claims of IO led to a reduced provision of abortions and abortion referrals in religious hospitals. Qualitative evidence also provided in-depth examples of how IO impacts clinical practice, including restricting standard practice for pregnancy complications and restricting referrals to other hospitals. Multiple studies also showed training of future abortion providers was impeded by IO. Given the overturn of *Roe v. Wade* is expected to significantly reduce abortion access in the United States, these findings suggest IO may further exacerbate local access issues.^61^ Studies from other parts of the world revealed that the use of IO was widespread and occurred at secular as well as religious institutions. Interestingly, the literature review did not find any evidence of positive impacts of IO, such as the potential benefits of protecting an institution’s moral integrity or identity. This finding may possibly reflect a bias towards negative impacts in the empirical literature or that there was insufficient evidence of positive impacts. Future empirical research exploring the positive impacts of IO warrants further exploration.

Second, if IO is morally justifiable, a further concern was the adequacy of each institution’s moral decision-making process. This was particularly pertinent to the review findings that some secular (including state) hospitals were claiming IO.^57–59^ The moral grounds on which IO was claimed in secular hospitals were not clear from the studies. This lack of transparency was concerning given IO conflicts with the duty of state hospitals to provide publicly-funded, legally available health services.^62^ Also relevant to the adequacy of the decision-making process was whether stakeholders should be involved in determining an IO. Biggs, Casas^50^ showed a lack of support from medical and midwifery students for universities to claim an IO. This raises the issue of whether institutions should require the consent of those affected before claiming an IO (in this case, students wanting to participate in abortion training).^63^ This leads to a broader question that if institutions can be moral agents, to whom are they morally responsible? Are they only responsible to the owners of the institution or to their stakeholders too?^63,64^ If the latter, then arguably students (in the case of universities), employees and patients (in the case of hospitals) should play a role in the decision-making process.

Third, the findings also revealed individual CO clauses could be misused to establish de facto IO where IO was not explicitly given legal protection. Evidence from qualitative studies demonstrated some new hospital employees were asked, and sometimes compelled, to sign forms claiming individual CO prior to commencement. An individual’s objection to abortion participation is only conscience-based if participating in the procedure is contrary to their core moral beliefs, and their refusal to participate is based on those beliefs.^2^ Signing an individual CO form should signify this choice, not the choice of their employer. Appropriate regulatory responses that prevent the misuse of employees’ individual CO as a mechanism for ensuring IO should be explored.

Fourth, the barriers IO poses to provision and receipt of legal abortion care require further research. As stated earlier, research on the impact of IO on patient outcomes is limited. In light of the barriers posed by IO, as shown in this review, there is a need for further evidence about the impact of IO on patients’ access to abortion services.

Finally, the findings suggest appropriate regulatory responses from governments are needed to balance the moral integrity of institutions with the needs of patients and employees and other stakeholders with divergent moral views. Our analysis showed that regulation of IO varied across countries. For example, in Colombia, there was case law expressly prohibiting IO, whereas in Chile, legislation protected IO but only for private institutions. In other countries, such as Australia, the law was silent, neither protecting nor prohibiting IO. More research is needed to ascertain the different forms of regulation of IO globally and determine their impact. Moreover, regulatory guidance (no matter what form it takes) should clarify: (1) if IO may be claimed; (2) under what conditions it can be claimed; and (3) how patients’ right to access abortion can be respected (and even facilitated) in light of IO. Legal options proposed to optimize the regulation of IO for other ethically-sensitive issues, such as voluntary assisted dying, may be useful for informing future approaches.^65^ For example, if IO is permitted, objecting institutions could be legally required to provide information about abortion, and facilitate referral to a willing institution or provider.^65^

#### Strengths and limitations

In this mixed-methods narrative review, we interpreted the empirical literature on IO, provided insights on key messages, and identified areas for future policy reform and further research. However, there were some limitations. First, only English language studies were included. Second, as the literature was screened by one author only, it is possible that some eligible studies were missed. Third, we relied on the description of IO laws and policies as described by the individual study’s authors, rather than sourcing the legislation or policies independently. This may have led to some inaccuracies or a lack of uniformity in how the regulations were described. Also, IO policies may have changed since an individual study were published. Finally, as this is not a systematic review, there is an increased possibility of bias in the interpretation of the literature. However, as the authorship team has combined expertise across the range of fields covered by this review (abortion provision, ethics and law), potential bias was minimized by drawing on diverse perspectives.

## Conclusion

Even though the ability of hospitals to claim an IO is morally contested, this review demonstrated that the practice occurred across a range of countries. Evidence from the United States demonstrated IO had a negative impact on clinicians providing and referring for abortions, and also restricted the training of future abortion providers. Outside of the United States, the findings showed IO was occurring at secular as well as religious institutions, that the decision-making processes leading to IO were often unclear and that individual CO clauses were sometimes misused to establish a de facto IO. Further research is needed on the moral justification for IO, how decisions to claim IO are made, and the grounds for claiming an IO. Appropriate regulatory responses are needed to ensure that protecting the moral integrity of institutions (by exempting them from abortion provision) is balanced with the needs of patients, employees and other stakeholders (such as medical students) who have divergent moral views. However, further research is needed to establish the optimal regulatory model.

## Declarations

**Ethics approval and consent to participate:** Not applicable because all data were from already published materials.

**Consent for publication:** Not applicable.

**Availability of data and materials:** All data and materials in the included studies are available open access or via subscription to the following academic databases: MEDLINE (Ovid), Embase (Ovid), CINAHL (EBSCO), Global Health (CAB Abstracts), ScienceDirect and Scopus.
